# Severe intracellular magnesium and potassium depletion in patients after treatment with cisplatin

**DOI:** 10.1038/sj.bjc.6601344

**Published:** 2003-10-28

**Authors:** H Lajer, H Bundgaard, N H Secher, H H Hansen, K Kjeldsen, G Daugaard

**Affiliations:** 1 Department of Oncology 5072, Rigshospitalet, University of Copenhagen, The Finsen Center, Blegdamsvej 9, Copenhagen DK-2100, Denmark; 2 Department of Cardiology, Rigshospitalet, University of Copenhagen, The Finsen Center, Blegdamsvej 9, Copenhagen 2100, Denmark; 3 Department of Anaesthesiology, Rigshospitalet, University of Copenhagen, The Finsen Center, Blegdamsvej 9, Copenhagen 2100, Denmark

**Keywords:** cisplatin, side effects, hypomagnesaemia, magnesium, potassium, depletion

## Abstract

The purpose of this study is (1) to evaluate skeletal muscle magnesium (Mg) and potassium (K) during treatment with cisplatin; (2) to evaluate the predictive value of plasma (P)-Mg for intracellular Mg during cisplatin treatment; and (3) to evaluate whether changes in intracellular K influence skeletal muscle Na,K-ATPase. In all, 65 patients had a needle muscle biopsy obtained before and 26 patients both before and after cisplatin treatment. Biopsies were analysed for Mg, K, and Na,K-ATPase concentrations, and P-Mg and P-K determined. Treatment with a total dose of ≈500 mg (270 mg m^−2^ surface area) cisplatin over 80 days was associated with reductions in muscle [Mg] (95% CI) (8.95 (8.23–9.63) to 7.76 (7.34–8.18) *μ*mol g^−1^ wet wt. (*P*<0.01), and muscle [K] (90.81 (83.29–98.34) to 82.87 (78.74–87.00) *μ*mol g^−1^ wet wt. (*P*<0.05), as well as in P-Mg 0.82 (0.80–0.85) to 0.68 (0.64–0.73) mmol l^−1^ (*P*<0.01 but not in P-K (4.0 (3.8–4.1) *vs* 3.8 (3.7–4.0) mmol l^−1^). No simple correlations were observed between P-Mg and muscle [Mg], or between P-K and muscle [K], either before (*n*=65) or after (*n*=26) treatment with cisplatin. The changes in [Mg] and [K] were not associated with changes in the muscle Na,K-ATPase concentration. Following treatment with cisplatin, an ≈15% decline in P-Mg was accompanied by an ≈15% loss of muscle [Mg], as well as an ≈10% reduction of muscle [K] and fatigue and muscle weakness previously ascribed to hypomagnesaemia may therefore also be well explained by muscle K depletion observed despite normal levels of P-K. There was no correlation between P-Mg and SM-Mg or between P-K and SM-K. Thus, P-Mg and P-K are not reliable indicators for Mg and K depletion during treatment with cisplatin. However, the majority of patients will present Mg and K depletion after cisplatin therapy and of these only very few patients will present a low P-Mg or P-K. Therefore, routine supplementation should be considered in all patients receiving cisplatin.

Cisplatin is one of the most important anticancer agents for solid tumours. Nephrotoxicity of cisplatin may result in increased magnesium (Mg) excretion (Mavichak *et al*, 1985; Stewart *et al*, 1985; Ariceta *et al*, 1997), even before renal function becomes affected (Daugaard *et al*, 1988). The lack of clear-cut symptoms in most patients with even profoundly reduced levels of plasma (P)-Mg has left it uncertain whether Mg substitution should be instituted when the value is low. Fatigue and muscle weakness are generally ascribed to hypomagnesaemia, although these symptoms can be found in many patients receiving noncisplatin containing chemotherapy as well. More feared, but rarely documented, symptoms associated with hypomagnesaemia are Torsade des pointes ventricular arrhythmia and muscular tetanus (Lajer and Daugaard, 1999).

Since only 0.3% of body Mg is present in the plasma and less than 1% is located in the extracellular space (Rude, 1993), the effect of cisplatin on the major but less-accessible Mg stores, or the relationship between P-Mg and these stores, during treatment with cisplatin remains unknown. Skeletal muscles account for ≈30% of body Mg, and since it is readily exchangeable, it is considered an important reservoir (Elin, 1987). Since Mg and potassium (K) concentrations correlate within skeletal muscles (Dorup *et al*, 1988b), the pathophysiology aspects of K metabolism become important if Mg depletion is suspected. K depletion is associated with a downregulation of Na,K-ATPase, which could explain fatigue and muscle weakness in cisplatin-treated patients.

The purpose of this study was (1) to evaluate skeletal muscle [Mg] and [K] during treatment with cisplatin; (2) to evaluate the predictive value of P-Mg for intracellular [Mg] depletion during cisplatin treatment; and (3) to evaluate whether potential changes in K metabolism influence skeletal muscle Na,K-ATPase.

## PATIENTS AND METHODS

A total of 128 patients scheduled to receive cisplatin-based chemotherapy were asked to enter the study and 68 patients accepted to participate. The study was approved by The Copenhagen Ethics Committee (KF 01-092/99), and was in accordance with Helsinki declaration II.

Muscle biopsies were planned before and following treatment. Depending on the treatment protocol, patients received 100 mg cisplatin intravenous (i.v.) m^−2^ body surface area once every 4 weeks (non-small-cell lung cancer (NSCLC)), 20 mg cisplatin i.v. m^−2^ every day for 5 consecutive days every 3 weeks (testicular cancer), or 40 mg cisplatin i.v. m^−2^ once a week for 6 weeks (cervix cancer). The average follow-up time from the start of treatment until the second biopsy was 12 weeks, and the average time from the last dose of cisplatin to the second biopsy was 4 weeks. All patients had performance status >2 according to the WHO criteria.

Patients with diabetes or thyroid/parathyroid diseases were excluded from the study to avoid the possible specific effects on Mg and K metabolism. No patient received thiazide diuretics, but a maximum of 40 mg of loop diuretics was administered every third/fourth week in connection with hydration therapy. No standard Mg administration was given, but three patients developed a P-Mg below 0.40 mmol l^−1^ once during the follow-up, and in these patients Mg was supplemented as a single i.v. infusion of 1000 ml isotonic glucose containing 50 mmol MgCl_2_. No K substitution was administered.

Patients received a mean cumulative prednisolone dose of 600 mg (range 150–900 mg) to reduce nausea, and standard intravenous hydration therapy consisted of 4–5 l of NaCl/glucose/mannitol per day on which cisplatin was administered.

The characteristics of the 65 patients who had successful pretherapy biopsy are presented in Table 1

**Table 1:** Clinical characteristics of patients

	**Before chemotherapy**	**Group with biopsies before: after chemotherapy**
Number of patients	65	26
Mean age in years (95% CI)	54 (51–57)	50 (45–55)
Male : female ratio	46: 19	16: 10
Mean interval between biopsies (95% CI)	—	80 (73–89)
Cancer location	141: 216: 35: 41: 51: 61	117: 27: 32
Mean weight before : after treatment in kg (95% CI)	—	73 (66–79): 72 (65–78), P=0.17
Mean ^51^Cr-EDTA clearance before : after treatment (ml (min.x1.73m2)^−1^) (95% CI)	—	91 (83–99): 74 (66–82), P<0.01
Mean cumulated cisplatin dose (mg) at the time of second biopsy (95% CI)	—	503 (435–572)
Mean cumulated cisplatin dose m^−2^ (mg) at the time of second biopsy (95% CI)	—	270 (236–304)

95% Confidence interval (CI) appears in brackets where indicated.

1 Non-small-cell lung cancer;

2 c. testis;

3 c. uterine cervix;

4 bladder cancer;

5 unknown primary tumour;

6 mesothelioma.

. The reasons for unavailability of both a pre- and post-therapy muscle biopsy (*n*=39) were patients declining from a second biopsy (*n*=15), logistic problems (*n*=6), death of patients (*n*=7), progression of disease where taking a second biopsy was found unethical (*n*=5), pretherapy biopsy (*n*=3) or post-therapy biopsy unsuitable for analysis (*n*=3). Unsuitable biopsies were biopsies too small for measurements to be made.

Characteristics of the 26 patients in whom paired samples were available are presented in Table 1. This group formed the basis of all analysis related to changes before and after chemotherapy. Glomerular filtration rate measured as ^51^Cr-EDTA clearance was reduced by 19% (*P*<0.01) after treatment, but no significant change in weight was observed.

In addition, it was decided at the start of the study to include patients, who could, for logistical reasons, not be included before therapy, with a single biopsy after therapy if they demonstrated hypomagnesaemia. This was done to obtain patients with P-Mg values in the lower range, thereby allowing a stronger testing for a possible correlation between P and muscle values within this range. Five such patients were included, and their demographic data were in accordance with those of the 26 patients depicted in Table 1.

### P-Mg and K

Blood samples for P-Mg and P-K were collected from a cubital vein. P-Mg was measured by the colorimetric end point method with xylidyl blue and buffer/EGTA using Roche/Hitachi 717 (Roche Diagnostics, Mannheim, Germany). P-K was measured by potentiometry (ion-selective electrodes) with valinomycin as ion-specific material using the same apparatus.

### Muscle biopsies

Biopsies of 20–30 mg wet wt. were obtained from the lateral vastus muscle, using a Bergström needle following local anesthesy of the skin, subcutis, and fascia (Lidocaine 1%) (Bergstrom, 1979). The biopsy was immediately dissected free of visible fat and connective tissue, divided and placed in sealed preweighted plastic tubes, and frozen at −80°C until measurements of K, Mg, and ^3^H-ouabain-binding sites. Measurements on specimens obtained from the same individual before and after cisplatin therapy were simultaneously performed.

In undersized muscle samples, measurements of Mg and K were given priority over ^3^H-ouabain binding.

### Skeletal muscle Mg and K

Skeletal muscle Mg (SM-Mg) content was measured in duplicate by atomic absorption (AAnalyst 100; Perkin-Elmer, Norwalk, CT, USA) at 285.2 nm (Dorup *et al*, 1988a; Bundgaard and Kjeldsen, 2002). The samples were dissolved in 1 ml of 30% H_2_O_2_, and the suspension was maintained at 90°C for 12 h to allow complete evaporation. After addition of 2 ml of trichloroacetic acid (TCA; 5% wt v^−1^), 0.5 ml of the solution was used for atomic absorption after final addition of a further 0.5 ml of 5% TCA and 1.5 ml of redistilled water. Skeletal muscle K (SM-K) content was measured by flame photometry with an FLM 3 (Radiometer, Copenhagen, Denmark) with lithium as an internal standard using a solution as used for atomic absorption, except that 1.5 ml of redistilled water was substituted by 5 mmol l^−1^ LiCl.

### ^3^H-ouabain binding

^3^H-ouabain binding in intact samples was performed as previously described (Kjeldsen, 1986; Bundgaard and Kjeldsen, 2002).

### Statistics

Values are means with 95% confidence intervals (CI) or median values with 25 and 75 percentiles as indicated. Calculations were performed using SPSS statistical software (SPSS Inc., Chicago, IL, USA). Differences in means between paired observations were calculated using paired *t*-test, except for the ouabain-binding site concentration where the sign test (nonparametric) was applied due to asymmetry in the difference between pairs. Correlations were evaluated using Pearson's test or, in case of non-normal distribution of data, the Spearman test. Linear regression analysis was carried out using the method of least squares. Regression lines with 95% mean prediction interval are shown. *P*-values <0.05 were considered statistically significant.

## RESULTS

In the 65 patients measured before cisplatin treatment (Table 1), P-Mg was 0.82 (0.80–0.84) mmol l^−1^, P-K 4.0 (3.9–4.1) mmol l^−1^, SM-Mg 8.95 (8.54–9.36) *μ*mol g^−1^ wet wt., and SM-K 94.77 (90.44–99.11) *μ*mol g^−1^ wet wt.

**Figure 1:**
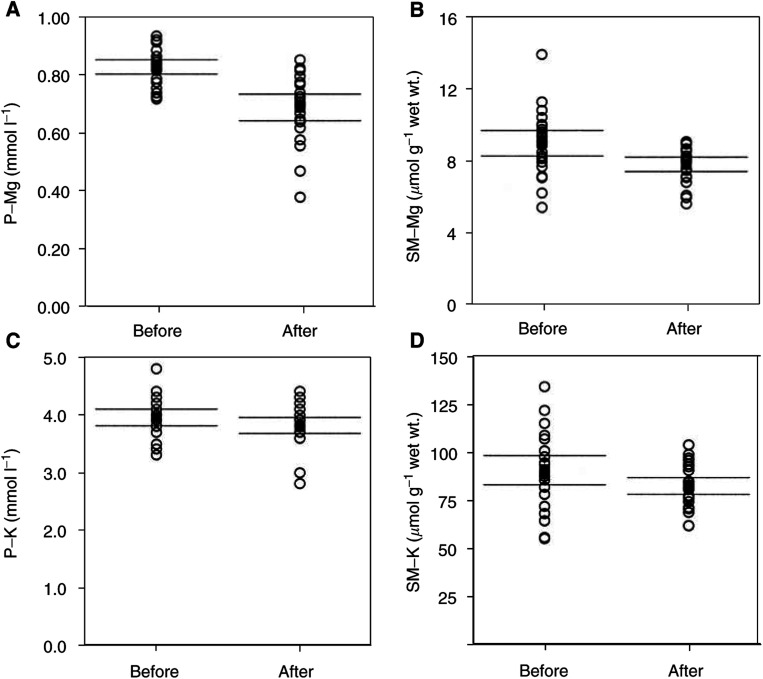
P-Mg (**A**), SM-Mg (**B**), P-K (**C**) and SM-K (**D**) before and after treatment with cisplatin. Rings represent individual patients. Black ring represents mean and lines 95% confidence interval of mean.

In the 26 patients with biopsies available before and after cisplatin treatment (Table 1), P-Mg fell from 0.82 (0.80–0.85) to 0.68 (0.64–0.73) mmol l^−1^ (*P*<0.01, mean difference 0.14 (0.09–0.19)); SM-Mg from 8.95 (8.23–9.63) to 7.76 (7.34–8.18) *μ*mol g^−1^ wet wt. (*P*<0.01, mean difference 1.19 (0.39–1.98)) and SM-K from 90.81 (83.29–98.34) to 82.87 (78.74–87.00) *μ*mol g^−1^ wet wt. (*P*<0.05, mean difference 7.94 (0.5–15.39), where as P-K did not change significantly (4.0 (3.8–4.1) to 3.8 (3.7–4.0) mmol l^−1^) (Figure 1

, *n*=26). Median values (25th and 75th percentiles) for ^3^H-ouabain-binding site concentration were also similar before and after treatment (263 (238–294) *vs* 266 (247–289) pmol g^−1^ wet wt.). The fraction of patients low in both P-Mg and SM-Mg increased from 11 to 41% following treatment with cisplatin. Using the lower CI from pretherapy values as reference, 58% of the patients were Mg depleted after treatment and out of these only one out of three presented with low P-Mg. Equally, 58% were K depleted and only 13% of these presented a low P-K.

**Figure 2:**
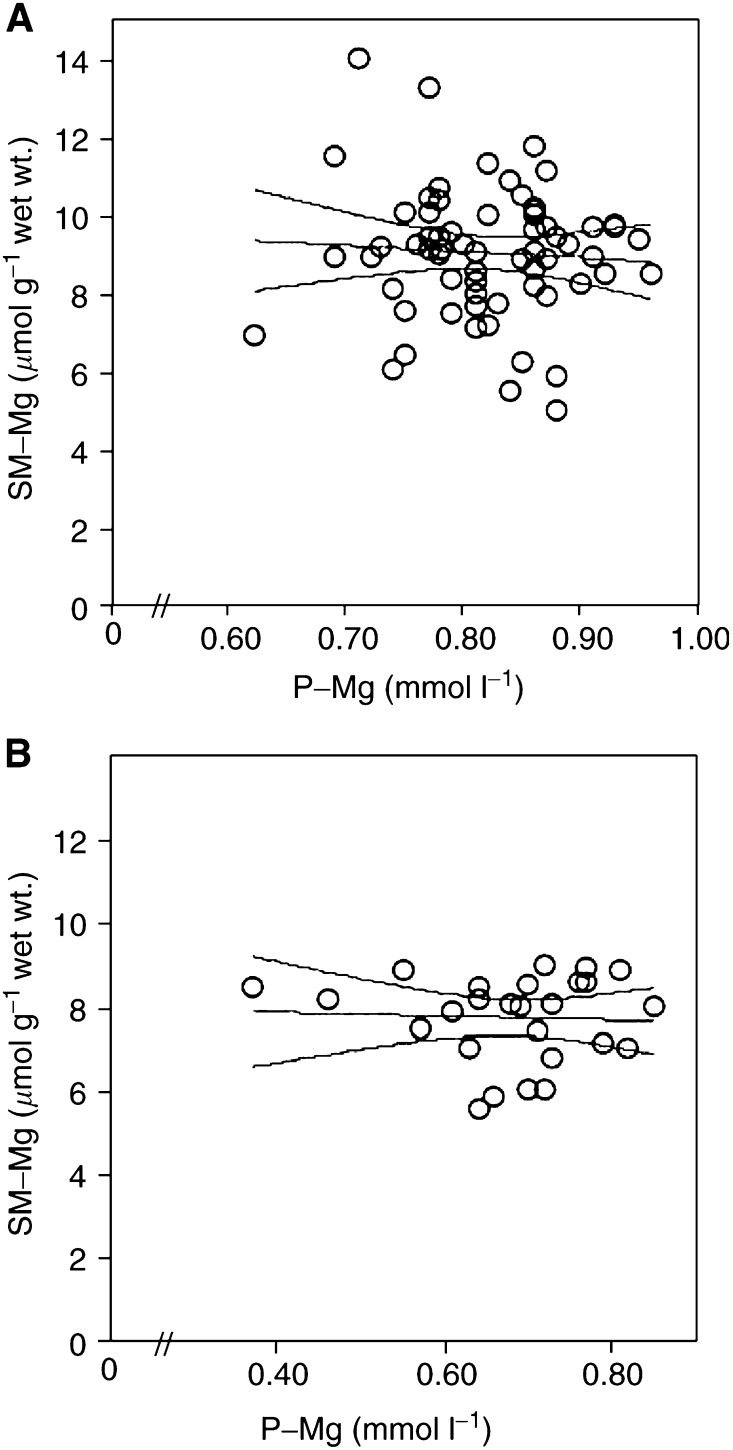
Associations between P-Mg and SM-Mg before (**A**) and after (**B**) chemotherapy. Rings represent individual patients. Centerline represents regression line and adjoining lines represent 95% confidence interval.

No significant association was observed between P-Mg and SM-Mg either before (*n*=65, *R*^2^=0.00) or after (*n*=26, *R*^2^=0.00) treatment with cisplatin (Figure 2

, *n*=65 (A) and 26 (B)). Subgroup analysis of patients with low P-Mg (<0.67 mmol l^−1^) following chemotherapy also showed no association between P-Mg and SM-Mg (*n*=10, *R*^2^=0.26). The same result was obtained if the analysis was carried out with the addition of the five patients included with hypomagnesaemia and the three patients in whom only a post-therapy biopsy was available, thereby increasing the number of patients with P-Mg <0.67 mmol l^−1^ (*n*=16, *R*^2^=0.02).

**Figure 3:**
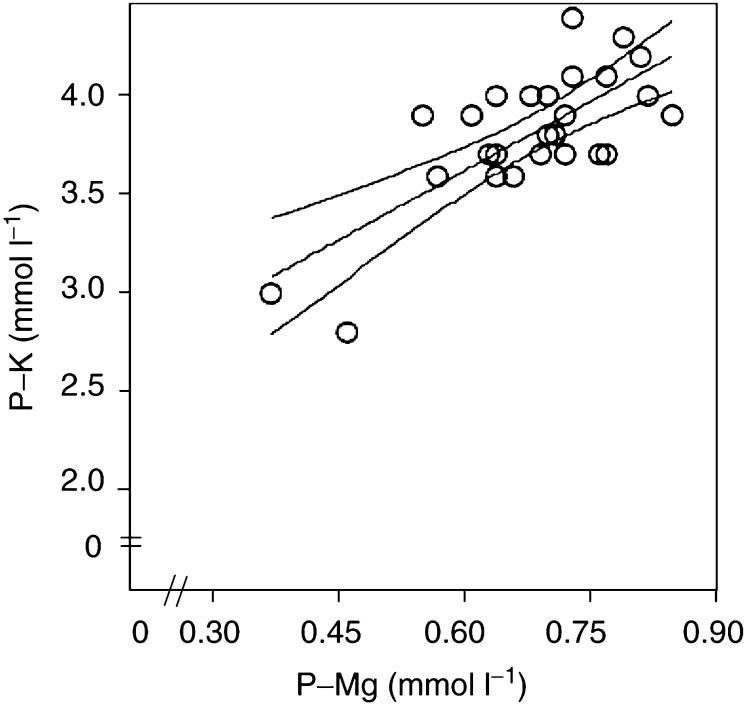
Association between P-Mg and P-K after chemotherapy. Rings represent individual patients. Centerline represents regression line and adjoining lines represent 95% confidence interval.

**Figure 4:**
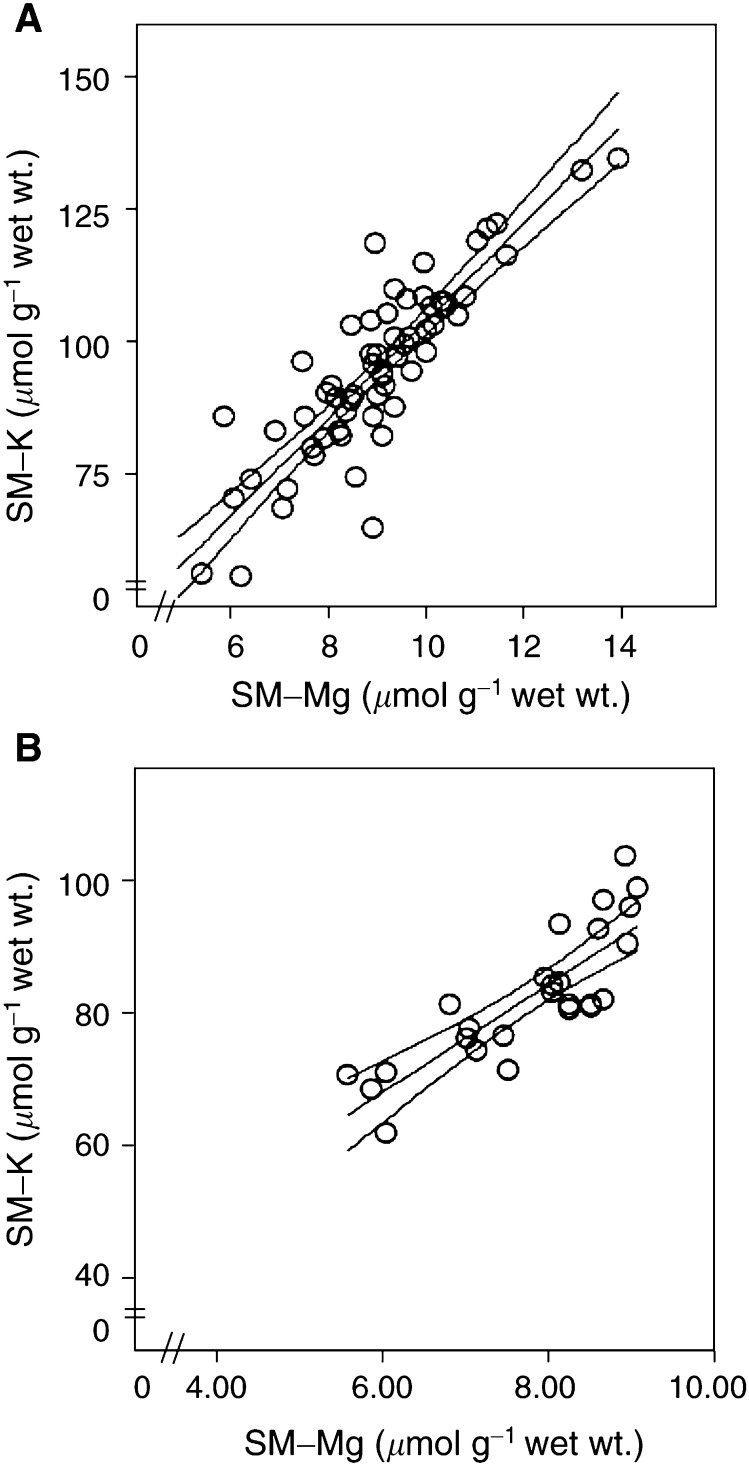
Associations between SM-Mg and SM-K before (**A**) and after (**B**) chemotherapy. Rings represent individual patients. Centerline represents regression line and adjoining lines represent 95% confidence interval.

**Figure 5:**
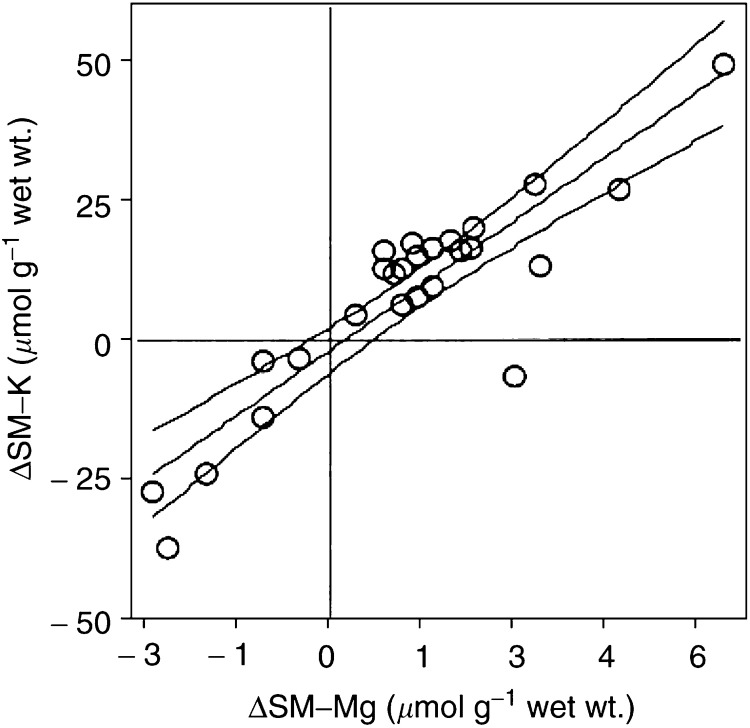
Association between changes in SM-Mg and SM-K during chemotherapy. Rings represent individual patients. Centerline represents regression line and adjoining lines represent 95% confidence interval.

Likewise, there was no significant association between changes in P-Mg and that in SM-Mg (*n*=26, *R*^2^=0.01), and there was no significant association between P-Mg and P-K before treatment with cisplatin, but such an association was observed after treatment (*R*^2^=0.55, *P*<0.01); (Figure 3

, *n*=26). An association was also observed between SM-Mg and SM-K both before (*n*=65, *R*^2^=0.76, *P*<0.01) and after (*n*=26, *R*^2^=0.70, *P*<0.01) treatment with cisplatin, as was a correlation between the change in SM-Mg and that in SM-K (*R*^2^=0.79, *P*<0.001; Figure 4A and B

, *n*=65 and 26, respectively, and Figure 5

, *n*=26). No association was observed between P-K and SM-K either before or after treatment, and equally there was no association between SM-K and skeletal muscle ^3^H-ouabain-binding site concentration either before or after treatment. Neither changed renal function, cumulated cisplatin dose nor age correlated to the observed changes in P-Mg, SM-Mg or SM-K.

## DISCUSSION

The concentrations of Mg, K and Na,K-ATPase observed before chemotherapy were in accordance with earlier studies, and therefore do not indicate an ion derangement in cancer patients when WHO performance status is better than 2 (Elin, 1987; Clausen, 1998). P-Mg was below the lower reference level after therapy in 38% of the patients and a novel observation was that the decrease in P-Mg was accompanied by an ≈15% loss of muscle Mg and ≈10% loss of muscle K; mean loss 1.2 and 8.0 mmol kg^−1^ wet wt. of skeletal muscle, respectively. If this observation represents all skeletal muscles (≈40% of the total body wt.), it would mean an average loss of ≈35 mmol of Mg and ≈225 mmol of K for a patient with a body weight of 70 kg, after administration of a cumulated dose of 500 mg cisplatin. Seven patients had a loss of as much as ≈25% of their Mg stores and three patients approached a 25% loss of their K stores. Since there was no simple correlation between P-values and the corresponding intracellular values, these severe losses would in clinical practice remain undiagnosed.

The observed depletion of skeletal muscle K during treatment with cisplatin was not suspected since a decline in P-K was not observed. The finding suggests that P-K is adjusted at the expense of intracellular stores during treatment with cisplatin. The K depletion questions whether K substitution is in demand in these patients or whether the K depletion can be expected to resolve following a normalisation in Mg stores. Furthermore, fatigue and muscular weakness ascribed to hypomagnesaemia may also be well explained by K depletion. After treatment with cisplatin, an association between Mg and K in plasma was observed, but this association was not present prior to therapy. This observation may be caused by a larger span of values after treatment, but could also indicate an association in the regulation of Mg and K in plasma under the present conditions.

Despite a decrease in skeletal muscle K, the Na,K-ATPase concentration was normal after cisplatin treatment. This is contrary to what was expected (Dorup *et al*, 1988b; Clausen, 1998), and may be caused by prednisolone administered as antiemetic to cisplatin-treated patients, since it reportedly upregulates the skeletal muscle Na,K-ATPase concentration (Thompson *et al*, 2001). Thus, prednisolone may prevent K depletion in cisplatin treatment from reducing muscle cell K reuptake capacity further. If hypokalaemia does develop in these patients, the potential for K uptake into skeletal muscle may therefore be high. This may aggravate hypokalaemia. Hypokalaemia in these patients could therefore be a more serious problem. On the other hand, Mg depletion may cause a functional reduction in Na,K-ATPase activity (Skou, 1965; Kjeldsen and Norgaard, 1987), which then induces depletion of K.

Treatment of hypomagnesaemia related to treatment with cisplatin has been directed towards normalisation of P-Mg with the secondary intention to restore total body Mg (Macaulay *et al*, 1982; Vokes *et al*, 1990; Evans *et al*, 1995). However, P-Mg is evidently not a reliable indicator of Mg depletion during cisplatin chemotherapy, and neither can Mg substitution be guided by this parameter alone. These arguments relate also to K. The approach towards substitution of the observed losses is therefore complicated, and it is not possible to develop simple guidelines for Mg or K substitution during treatment with cisplatin. Furthermore, it remains unknown whether Mg and K substitution is effective in increasing body Mg and K stores during cisplatin treatment, especially in the presence of a cisplatin-induced renal reabsorption defect.

In conclusion, cisplatin treatment caused significant Mg and K depletion in the majority of patients and, in most patients, this depletion was observed despite normal corresponding *P* values. Therefore, due to our inability to monitor these electrolytes during treatment, it should be considered whether routine Mg and K supplementation should be implemented in these patients from the start of treatment.
